# Permeability and ammonia selectivity in aquaporin TIP2;1: linking structure to function

**DOI:** 10.1038/s41598-018-21357-2

**Published:** 2018-02-14

**Authors:** Viveca Lindahl, Pontus Gourdon, Magnus Andersson, Berk Hess

**Affiliations:** 1 Department of Physics and Swedish e-Science Research Center, KTH Royal Institute of Technology, Science for Life Laboratory, Stockholm, Sweden; 2 Department of Biomedical Sciences, University of Copenhagen, Copenhagen, Denmark; 3 Department of Experimental Medical Science, Lund University, Lund, Sweden

## Abstract

Aquaporin TIP2;1 is a protein channel permeable to both water and ammonia. The structural origin of ammonia selectivity remains obscure, but experiments have revealed that a double mutation renders it impermeable to ammonia without affecting water permeability. Here, we aim to reproduce and explain these observations by performing an extensive mutational study using microsecond long molecular dynamics simulations, applying the two popular force fields CHARMM36 and Amber ff99SB-ILDN. We calculate permeabilities and free energies along the channel axis for ammonia and water. For one force field, the permeability of the double mutant decreases by a factor of 2.5 for water and 4 for ammonia, increasing water selectivity by a factor of 1.6. We attribute this effect to decreased entropy of water in the pore, due to the observed increase in pore–water interactions and narrower pore. Additionally, we observe spontaneous opening and closing of the pore on the cytosolic side, which suggests a gating mechanism for the pore. Our results show that sampling methods and simulation times are sufficient to delineate even subtle effects of mutations on structure and function and to capture important long-timescale events, but also underline the importance of improving models further.

## Introduction

Aquaporins are a family of transmembrane proteins found in cells of all types of organisms, from eukaryotes to bacteria and archaea. They specifically and efficiently regulate the transport of fluids between cells or organelles, a function fundamental for all life. In humans more than ten different aquaporins are expressed in the body, each with its own specific function. These nano-sized pores reside in the hydrophobic membrane where they provide a path for water to efficiently pass through. Several aquaporins are in addition selectively permeable to certain solutes such as glycerol, hydrogen peroxide, carbon dioxide and ammonia^1^. The finely tuned physical properties of aquaporins combined with their high biological relevance has inspired a massive amount of research in both theoretical and experimental biology and physics with applications ranging from drug design^2^ to nanotechnology^3^.

**Figure 1:**
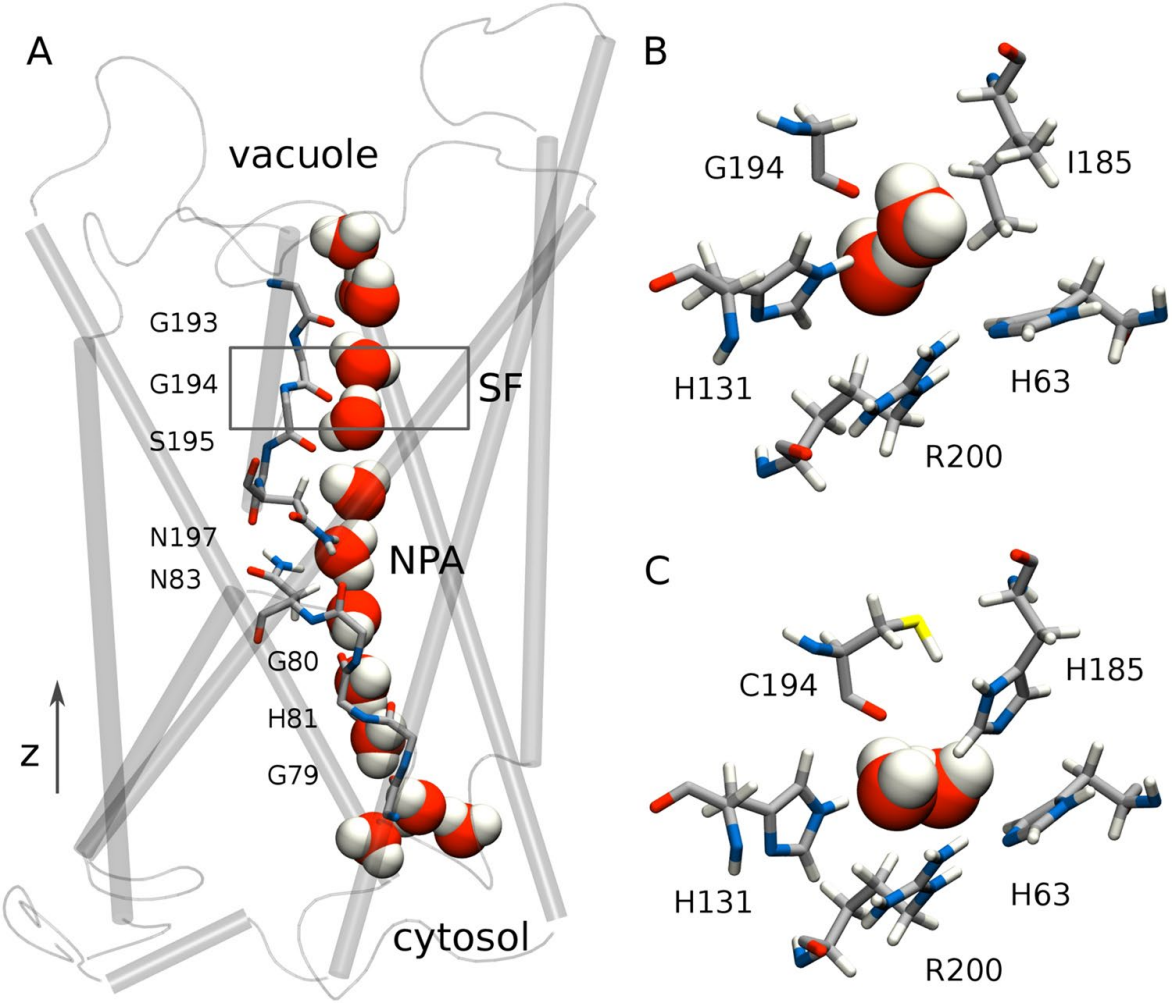
TIP2;1 structure. (**A**) The TIP2;1 monomer (side view). Backbone carbonyl oxygens and two asparagines in the central NPA region provide hydrogen bonding sites for the line of water molecules filling the pore. The direction of the channel axis *z*, going from the cytosolic side (bottom) to the inside of the vacuole (top), is indicated. (**B**) SF composition of TIP2;1 (top view). (**C**) SF composition of the double mutant I185H × G194C (top view). The shown structures are from arbitrarily chosen, equilibrated trajectory frames.

Structurally, aquaporins form tetramers, where each monomer acts as a functional pore^4^. The inside of the pore is, perhaps surprisingly, hydrophobic with the exception of a few distinct sites. From top to bottom, it is lined with backbone carbonyl oxygens which accept hydrogen bonds from the water molecules, see Fig. 1A. At the center of the pore, asparagines of two highly conserved so-called NPA-motifs instead donate hydrogen bonds to water which helps stabilize the bipolar orientation of the water chain^5^.

Solute selectivity of the channel is highly sensitive to the composition of the selectivity filter (SF), a set of four amino acid sites located at the entrance of the pore. Typically the SF is the narrowest region of the pore and so can sterically block passage of solutes significantly larger than water. For narrow pores, where the water molecules filling the pore align close to single-file, polar SF residues may act as a filter against hydrophobic molecules. In the case of human AQP1, which is generally reported to be water-only permeable, the highly conserved SF arginine has been argued to be the key residue in such a mechanism^6^.

Clearly, the more similar the solute is to water the more challenging it becomes to achieve high selectivity without also affecting water permeability. Ammonia (NH_3_) selectivity is a particularly interesting case since it is a polar molecule of similar size and dipole moment as water. One expects ammonia to be able to fit through narrow constrictions just like water and to readily hydrogen bond both to water and to polar sites of the pore. Indeed, both mammalian and plant aquaporins have been reported to be permeable to ammonia, at least to some degree, based on experiments using expression systems of yeast or egg cells of frog^1,7^.

An example of an ammonia-permeable aquaporin is TIP2;1 from the wheat plant^1^ found in the membrane surrounding the vacuole organelle of the cell. Interestingly, it shares the SF composition H63, I185, G194, R200 (using here the residue ids of TIP2;1), see Fig. 1B, with another ammonia permeable aquaporin, the mammalian AQP8. Up until recently however, there was no crystal structure available for either protein, hindering atomic-level studies. The first crystal structure of TIP2;1^8^ revealed an unexpected position of the SF arginine R200 where it instead of offering hydrogen bonds into the pore is occupied in hydrogen bonding with the neighboring histidine, H63. It was also suggested based on this structure that the SF should be extended to include a fifth residue, H131, with the motivation that it sterically enforces the special position of R200 and that it interacts with water molecules entering the pore.

Experiments on yeast mutants concluded that out of the mutations in the SF of TIP2;1 that mimic that of AQP1, namely H63F, I185H and/or G194C, two modified the ammonia permeability of TIP2;1^8,9^. Yeast expressing H63F or the double mutant I185H × G194C failed to grow, consistent with a loss of ammonia permeability. The double mutant SF is shown in Fig. 1C. The single mutant G194C had no effect, while I185H was reported to reduce growth in one study^9^ and in the other not^8^. The results for TIP2;1 and the double mutant were also confirmed in experiments using frog egg cell expression systems^10^.

To understand TIP2;1 permeability and selectivity, the outcomes of these experiments need to be explained on the atomic level. Information of such resolution can be obtained from molecular dynamics (MD) simulations. Aquaporins are already well-known figures in the world of MD simulations, but since the pioneering simulations^11,12^ feasible trajectory lengths increased by several orders of magnitude and now span the microseconds timescale. Here we investigate the origin of ammonia selectivity and the determinants of permeability by performing microsecond long MD simulations of TIP2;1, the double mutant I185H × G194C and the two corresponding single mutants. In order to gauge the strength of our simulation results, we apply two different force fields: CHARMM36 and Amber ff99SB-ILDN. We calculate permeabilities and free energy profiles for water and ammonia along the pore. Free energies for ammonia are calculated using an adaptive biasing method, the accelerated weight histogram method (AWH), which ensures that in each monomer an ammonia molecule overcomes free energy barriers and permeates, in a stochastic way, through the pore multiple times within one simulation. We observe that the mutations influence both permeabilities and free energies, and discuss the structural origin of these changes. In addition, for one force field, we see the pore transitioning between an open and a closed state, supporting the presence of TIP2;1 gating.

## Results

In the following we present results from 1 *μ*s long simulations of four different systems: wild type TIP2;1, in the following referred to as simply TIP2;1, G194C, I185H and I185H × G194C, applying both CHARMM and Amber force fields. For some systems and simulation setups we generated two trajectories. A summary of the data set is presented in Supplementary Table S1.

Generally, observable averages are calculated using data from all monomers of the system, from multiple simulations if there is more than one of the same system. The uncertainty of an average *μ* is given as the mean square error $\pm {\sigma }_{\mu }=\sigma /\sqrt{n}$, where *σ* is the standard deviation across monomers and *n* is the number of monomers included in the average *μ*.

We exclude data for monomers in which there are sudden disruption of hydrogen bonds and structural changes in the highly conserved NPA region. Since we expect the core of the channel to be stable, we consider it most likely that these events arise from modeling issues, such as the force field in combination with the starting structure. Excluding these rare events ensures that our results are reliable for timescales shorter than the simulation time. There are four hydrogen bonds in the NPA region that are present in the crystal structure and stabilize the orientation of the NPA motifs: two between the loops of each motif, N197–V82 and N83–M196, and two within each motif, N83–A85 and N197–A199. These are typically stable throughout the simulation, but for some monomers in our CHARMM simulations the N197–V82 hydrogen bond suddenly breaks and then often does not reform during the rest of the simulation. The following structural changes tend to be accompanied by disordered water chains and/or a sudden drop in permeability. In terms of minimum distances between these four NPA residue pairs, here we only include data from monomers for which all distances <4 Å for 90% of the trajectory. For the CHARMM data set this excludes up to one monomer per *μ*s-long trajectory, and for Amber, none. We refer to Supplementary Table S1 for a list of the simulations and excluded monomers, if any.

### Water distribution and interactions

**Figure 2:**
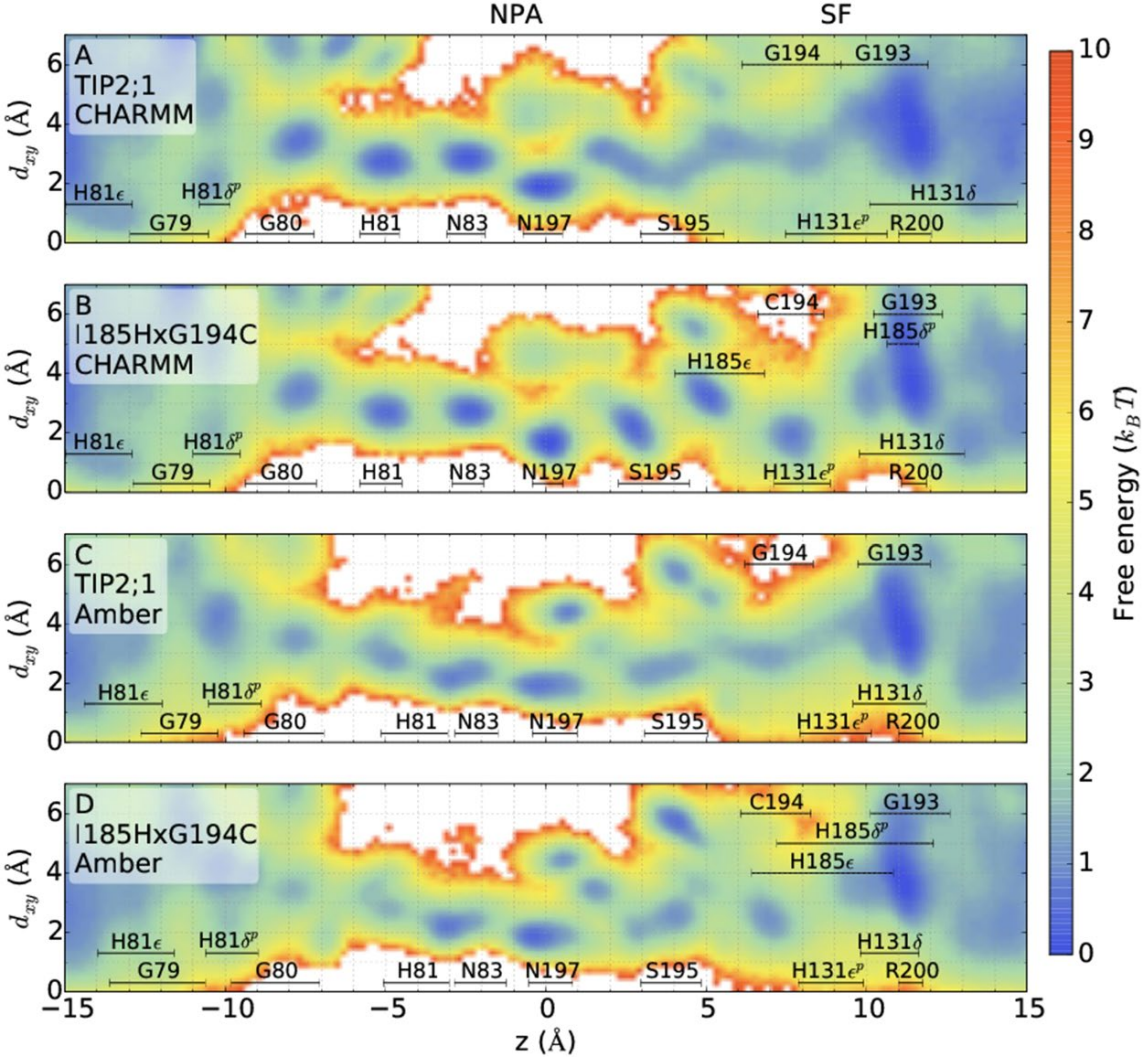
Free energy of water positions as a function of the distance along *z* and the radial distance from the channel center of mass, *d*_*xy*_. The free energies were calculated from water densities in unbiased simulations. Average *z* positions (±the standard deviation) of water molecules when hydrogen bonding to pore sites are indicated. The positions of the labels along the *y*-axis are arbitrary (do not indicate *d*_*xy*_ position). For the histidines, both N_*δ*_ and ${{\rm{N}}}_{\epsilon }$ sites are included separately. The protonated nitrogen is indicated by superscript *p*. See Supplementary Fig. S1 for the free energies of the single mutants.

Before adding the complexity of ammonia, we first try to better understand water by itself in the pore by studying the distribution and interactions of water and how these are affected by the SF mutations. The free energy as a function of the channel axis *z* and the radial distance from the *z*-axis shows the preferred locations of water molecules, see Fig. 2 for the cases of TIP2;1 and the double mutant, for both force fields. Results for the single mutants are included in Supplementary Fig. S1. To orient ourselves, the figure also indicates the locations of water molecules hydrogen bonding to the main pore lining sites: the array of carbonyl oxygens (residues G79, G80, H81, G193, S195, G194), the N_*δ*_ donors of the NPA asparagines (N83, N197) and the SF histidine site(s) ${N}_{\epsilon }$, *N*_*δ*_ (H131, H185). Note that the SF residue H63 is not included since it mainly is locked into hydrogen bonding with R200, the highly conserved SF arginine, which for the same reason interacts strongest with water outside and not within the constriction of the pore.

We begin by looking at results for CHARMM and then compare to Amber. The free energy landscape for TIP2;1 (Fig. 2A) in the SF region is relatively flat with wide minima and low barriers in between. In contrast, from the NPA region and below there are four well-defined minima separated by distances of 2.5 Å and by barriers of height 1–2 *k*_*B*_*T*. In each of these minima, water interacts with a single pore site.

The double mutation I185H × G194C (Fig. 2B) has a dramatic “sharpening” effect on the upper part of the free energy landscape, making it look very similar to the bottom part. There are now three distinct minima above the NPA region, separated by 2.5 Å, in which water interacts mainly with either of S195, H185 or C194 and H131.

**Figure 3:**
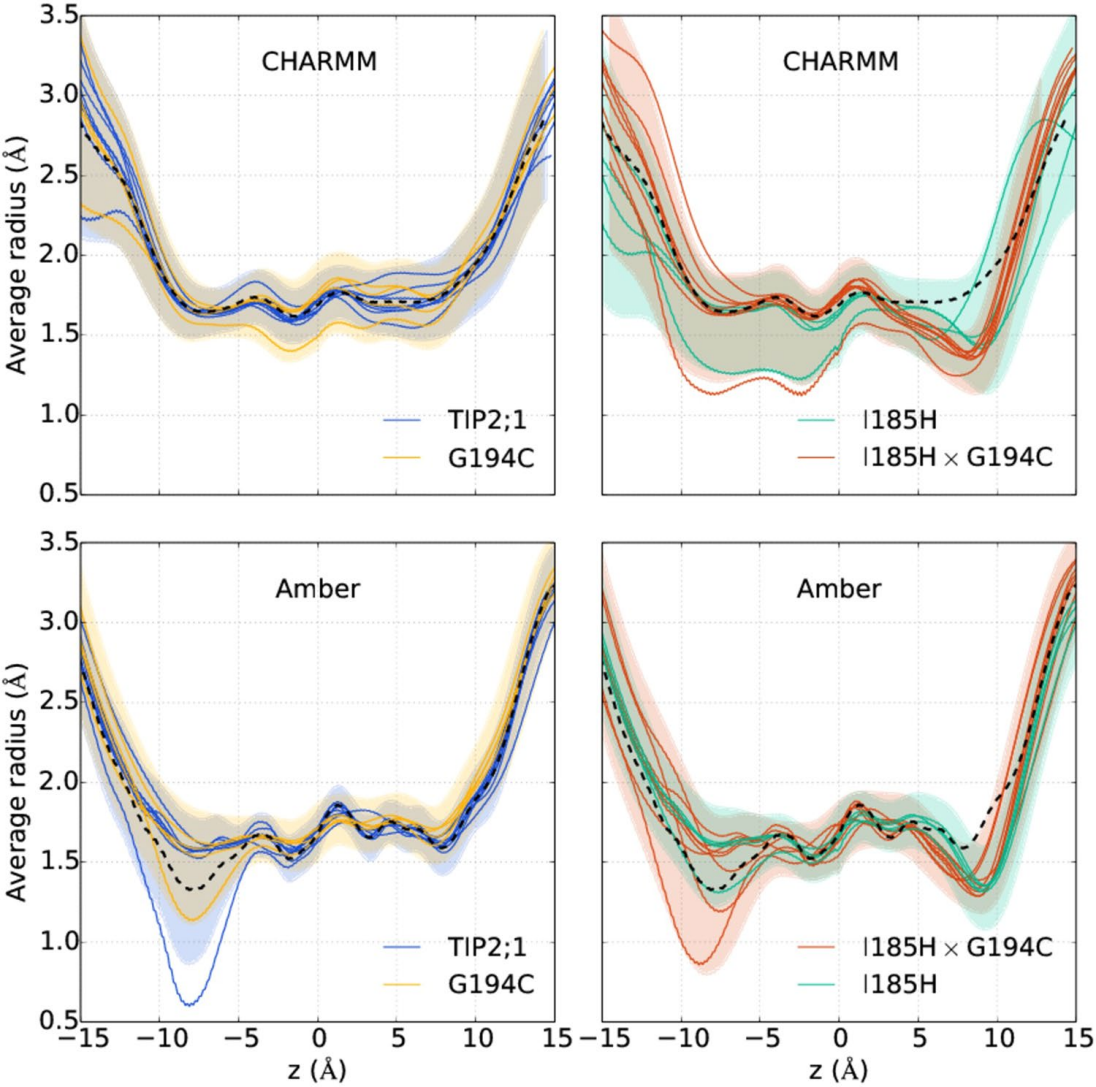
Average pore radius of TIP2;1 and mutants as a function of the channel axis *z*. Each profile is obtained by averaging over 1 *μ*s long trajectories. Different profiles of the same color correspond to different monomers of the same system. The dashed, black profile of each panel is the average over monomers for TIP2;1 (for the respective force field). For certain systems, there is data from two simulations yielding eight profiles in total. Error bars show the root-mean square deviation as a function of *z* for all trajectories of the same system, concatenated. For a few monomers of CHARMM, structural changes involving the NPA region (discussed in the main text) are evident from the decrease in radius in the bottom part of the pore. For Amber, in certain monomers a structural change involving H81 closes the pore on the cytoplasmic side, leading to a local decrease in pore radius.

We can understand the difference between TIP2;1 and the double mutant as a result of a few cooperating factors. Firstly, the bulky histidine of the I185H mutation narrows down the constriction of the pore. In the SF region, ${d}_{xy}\mathop{ > }\limits_{ \tilde {}}3$ Å is accessible to water in TIP2;1, but is partially blocked off by H185 in the double mutant, cf. Fig. 2A,B. In terms of the average pore radius, the same effect is seen as a local decrease from 1.8 to 1.4 Å at *z* = 8 Å, see Fig. 3 for average profiles together with root-mean-square deviations. This added constriction additionally forces water to pass closer to and interact with H131 located on the opposite side of the SF, cf. Fig. 1B,C. In fact, the probability of a water molecule to hydrogen bond to either the SF sites C194:O and H131:${{\rm{N}}}_{\epsilon }$ when located in the SF region increases by a factor 2–3 for the double mutant, and the probability of hydrogen bonding simultaneously to both increases by a factor of 4, see Table 1.

**Table 1:** Probabilities per water molecule of hydrogen bonding to SF sites (%).

System	G194:O	H131:${{\bf{N}}}_{{\boldsymbol{\epsilon }}}$	Both
*CHARMM*			
TIP2;1	25 ± 4	13 ± 4	6 ± 2
G194C	33 ± 2	18 ± 3	9 ± 2
I185H	15 ± 3	12 ± 6	4 ± 2
I185H × G194C	52 ± 2	37 ± 4	25 ± 3
*Amber*			
TIP2;1	48.2 ± 0.7	16.2 ± 0.9	7.5 ± 0.6
G194C	42 ± 7	14 ± 3	7 ± 2
I185H	42 ± 2	18.5 ± 0.9	7.0 ± 0.4
I185H × G194C	55 ± 3	15 ± 1	7.3 ± 0.8

The given values are probabilities of a water molecule located in the SF region, here defined as 6 Å < *z* < 9 Å and *d*_*xy*_ < 7 Å, to hydrogen bond to either of the listed pore sites, or to both.

Secondly, H185 in the double mutant is oriented with its aromatic ring parallel to the channel axis such that H185:${{\rm{N}}}_{\epsilon }$ provides a new hydrogen bonding site for water located fairly deep inside of the pore where it has potential to influence the whole upper half of the water chain. In TIP2;1, a water molecule in this region (4.0 Å < *z* < 6.8 Å) lies in between pore residues S195, G194 and H131 and has on average 0.36 ± 0.04 hydrogen bonds to either of them. In the double mutant this number increases to 0.79 ± 0.03, by roughly a factor of 2. The average number of water–water hydrogen bonds per molecule in this interval is however not affected: we obtain 0.96 ± 0.08 TIP2;1 and 0.98 ± 0.04 for the double mutant. Overall, more pore–water interactions in a narrower pore favors energetic rather than entropic contributions to the free energy which is consistent with the shift from a smooth to a rugged free energy landscape in TIP2;1 versus I185H × G194C.

**Figure 4:**
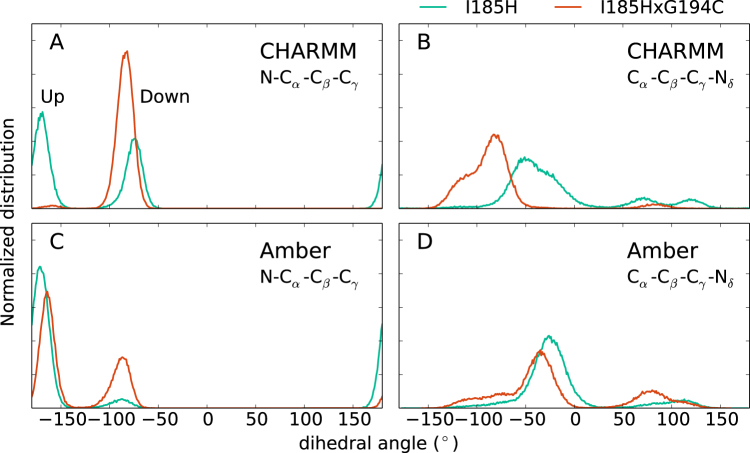
Distributions of two H185 dihedral angles. The N-C_*α*_-C_*β*_-C_*γ*_ dihedral (**A**,**C**) corresponds to moving the H185 aromatic ring further out of (up) or into (down) of the pore, while C_*α*_-C_*β*_-C_*γ*_-N_*δ*_ (**B**,**D**) represents a rotation of the aromatic ring. The distributions are different for the single mutant (turquoise) and double mutant (red), indicating the effect of the G194C mutation. Each histogram is based on one 1 *μ*s long trajectory. See Supplementary Fig. S2 for the corresponding trajectories.

From the previous reasoning, it might seem that the greatest effect of the double mutation I185H × G194C arises from the I185H substitution and thus one might conclude that the single mutant I185H should be very similar to the double mutant. However, it turns out that the G194C mutation is important for stabilizing the position of the H185 side chain. Due to the sulphydryl group in the cysteine sidechain, G194C locks the ring into place and sterically prevents it from flipping. Without this substitution the H185 sidechain is more mobile. For the single mutant I185H, the intervals along *z* where water interacts with the ${{\rm{N}}}_{\epsilon }$ and N_*δ*_ site of H185 are wide and overlapping and the free energy landscape is relatively smooth (see Supplementary Fig. S1). The impact of G194C can also be seen in the distribution of H185 dihedral angles, see Fig. 4. The angle N-C_*α*_-C_*β*_-C_*γ*_, see Fig. 4A, has two clear states, “up” and “down”, in which the histidine ring is orientated more out of or into the pore, respectively. The single mutant I185H transitions between these two states while the double mutant essentially only samples the down state. In addition, the dihedral angle C_*α*_-C_*β*_-C_*γ*_-N_*δ*_, see distributions in Fig. 4B, quantifies a “twist” of the aromatic ring. In the single mutant the distribution is wider and shifted closer towards 0° which corresponds to the ring lying nearly orthogonal to the channel axis.

Also by itself, the single mutant, G194C induces a subtle but measurable structural change. The added bulkiness of cysteine causes a small local shift of the backbone which brings the carbonyl oxygen of C194 to within hydrogen bonding distance of H131 (for instance, the average minimum distance between these residues decreases from 4.6 ± 0.3 to 3.2 ± 0.1 Å). This reorientation makes it slightly more favorable for a water molecule to interact with the pore in the SF region, see Table 1.

The Amber simulations show qualitative similarities to CHARMM but also clear differences. Also here, the free energy of TIP2;1 is smoother above than below the central NPA sites (Fig. 2C) and the double mutation increases barriers in the SF region (Fig. 2D). However, the properties of H185 are quite different for Amber and CHARMM. With Amber, H185 interacts with water further up in the pore, cf. Fig. 2B,D. Consistently, the histidine ring samples both the up and down state of the dihedral angle mentioned previously, see Fig. 4C. In fact, we see from the distributions of these dihedral angles that the effect of using a different force field may be larger than the effect of applying a mutation for a single force field. For instance, in terms of these distributions the Amber double mutant is more similar to the CHARMM single mutant I185H than to the CHARMM double mutant. We also note that the hydrogen bonding network in the SF region is different for Amber and CHARMM, see Table 1. For Amber TIP2;1, water interacts a factor of 2 more with G194:O and the effect of the mutations is weak.

### Water permeabilities

To measure the effect of the mutations on the pore function, we calculated water permeabilities. The diffusion permeability *p*_*d*_ is proportional to the number of permeation events per unit time in the simulation. The osmotic permeability *p*_*f*_ on the other hand, is proportional to the “hopping rate” of the water chain in a single-file model^13^.

The permeabilities were calculated from data where the pore is open. For Amber, at least once per simulation the pore is blocked on the cytosolic side by H81, see more details and discussion in the corresponding section below. Such closing events could be relevant for the function of the pore, e.g. as a gating mechanism, but for this analysis we are mainly interested in understanding the effect of mutations on permeability.

For CHARMM, *p*_*f*_ ≈ 5.3 ± 0.6 10^−14^ cm^3^ s^−1^, see Table 2, which is a factor of 5 lower than the value reported in^8^, *p*_*f*_ ≈ 25 ± 4 10^−14^ cm^3^ s^−1^ (where the uncertainty was given as the standard deviation across monomers), despite the fact that we use a very similar simulation setup (i.e. force field and MD settings) and the same method of calculating *p*_*f*_. To test the calculation method, we varied the definition of the pore, in terms of radius and length, and tested decreasing the time step between frames (from 50 ps to 5 ps) but these variations could not account for the discrepancy. To test for model differences, we made our models even more similar to that of^8^ by removing the use of virtual sites and using a 2 fs MD time step. But for a 200 ns TIP2;1 simulation, we saw no significant effect on *p*_*f*_. Nonetheless, our *p*_*f*_ and *p*_*d*_ values are consistent with each other. The ratio *p*_*f*_/*p*_*d*_ can be interpreted as a measure of the effective number of steps a water molecule needs to take to permeate the channel. In our case this ratio is 5–6 for all systems, which seems consistent with the number of free energy minima in the pore (Fig. 2) and with ratios calculated for other aquaporins^14,15^.

**Table 2:** Water permeabilities.

System	***p***_*f*_	*p*_*d*_
*CHARMM*		
TIP2;1	5.3 ± 0.6	1.0 ± 0.1
G194C	4.7 ± 0.2	0.86 ± 0.02
I185H	3.0 ± 0.5	0.6 ± 0.1
I185H × G194C	2.0 ± 0.1	0.41 ± 0.04
*Amber*		
TIP2;1	11.9 ± 0.6	1.99 ± 0.09
G194C	10.7 ± 0.1	1.92 ± 0.02
I185H	8.8 ± 0.4	1.59 ± 0.05
I185H × G194C	7.5 ± 0.3	1.37 ± 0.06

*p*_*f*_ and *p*_*d*_ are given in units of 10^−14^ cm^3^ s^−1^.

For both force fields, we see the same trend in the permeability for the mutations: TIP2;1 > G194C > I185H > I185H × G194C. In absolute terms, the values for Amber are a factor of 2–3 larger than for CHARMM, but relative values are more consistent. For G194C, the effect is small but there is a decrease by up to 10% relative to TIP2;1. I185H induces a larger decrease of 30–40%. The double mutation decreases the permeability the most, by 60% for CHARMM and up to 40% for Amber. For CHARMM, there is clearly a cooperative effect between the two mutations.

The decreased permeability caused by I185H can be explained by the resulting smaller pore radius and additional polar sites. The effect of G194C further shows that the permeability is sensitive to the exact orientation, and possibly mobility, of the pore sites. Analogous results were obtained in MD simulations of other aquaporins where narrower, more polar and more selective pores were also less permeable^14^. It is also in line with experimental *p*_*f*_ measurements indicating that permeability strongly depends on the number of accessible hydrogen bonding sites in the pore^16^. However, experiments measured essentially the same permeabilities for TIP2;1 and I185H × G194C^10^. Thus, some inconsistencies still remain between experiments and simulations^14,16^.

### Ammonia selectivity

Experimentally, the double mutant is known to substantially decrease ammonia permeability. We would expect such an effect to be detectable in terms of increased free energy barriers. Thus, to probe differences in ammonia selectivity among the mutants we calculated free energies for ammonia along the channel axis *z*. For this, we performed separate simulations with ammonia present, one molecule per monomer.

The free energy was calculated using the adaptive biasing method AWH, described in more detail in sec:methods. Briefly, the position along *z* of each ammonia molecule is controlled by coupling it to a harmonic bias potential centered at a value *λ*, such that *z* ≈ *λ* is enforced. For the narrow pore of aquaporin, using fixed restraints as is done in more traditional sampling methods, can however block important rearrangements of pore lining side chains or the solute itself. Deterministically moving restraints may give rise to nonequilibrium artifacts^17^. With AWH, *λ* is rather a dynamic variable *λ*(*t*) that is driven, in a stochastic manner, to converge to the given target distribution (here chosen uniform for |*z*| < 20 Å). The whole channel axis is thus traversed multiple times, within a single simulation. The free energy is estimated simultaneously and as it converges, the dynamics of *λ* becomes increasingly diffusive, following the intrinsic dynamics of the system. E.g. in the AWH simulations using CHARMM, the rate for ammonia to cross the pore was 29 *μ*s^−1^ for TIP2;1 and 12 *μ*s^−1^ for the double mutant, in line with the mutant having a lower water permeation rate (Table 2). To our knowledge this is the first time an adaptive biasing method has been applied to enhance sampling along an aquaporin channel (without partitioning the sampling into disjoint sampling windows^17^).

**Figure 5:**
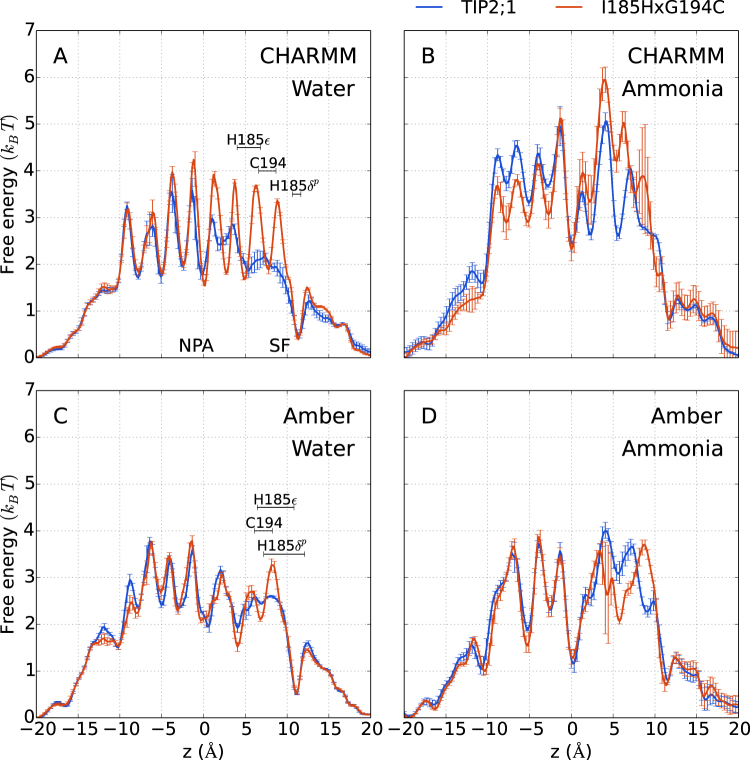
Free energies along *z* for ammonia and water. The error bars indicate estimated uncertainties of the average taken over the monomers. The larger error bars at *z* ≈ 4 Å in the profile for ammonia and Amber I185H × G194C are due to the rare sampling of a side pocket in one of the monomers. For the ammonia profile of CHARMM I185H × G194C, the uncertainty of the barrier height at *z* ≈ 9 Å is large due to H131 flipping away from the SF region in one of the monomers, decreasing the free energy estimate. The location of mutated residues are indicated by the average *z* positions (± the standard deviation) of water molecules when hydrogen bonding to these pore sites (as in Fig. 2).

For CHARMM, the double mutation increases the maximum free energy barrier for ammonia by 1 *k*_*B*_*T*, see Fig. 5B, indicating that the double mutation disfavors permeation of ammonia. The largest change in free energy occurs at the *z* ≈ 5 Å minimum where H185:${{\rm{N}}}_{\epsilon }$ provides an additional pore interaction site in the double mutant. For the CHARMM single mutants we observe no increase of barriers (see Supplementary Fig. S1), and similarly for the Amber double mutant, see Fig. 5D (for Amber single mutants, data with ammonia was not generated). Since the free energy barriers for ammonia only change significantly for the CHARMM double mutant, we continue by analyzing this case more closely.

To study selectivity of ammonia relative to water, we also computed one-dimensional free energy profiles of water, see Fig. 5A. Assuming that the permeability is proportional to ${e}^{-{F}_{b}/{k}_{B}T}$, where *F*_*b*_ is the maximum barrier height relative to bulk water, the free energy profiles predict a reduced permeability in the double mutant by a factor of 2.6 for ammonia and 1.9 for water. This corresponds to a small increase of selectivity for water by a factor of 2.6/1.9 = 1.4. The fact that the actual change in water permeabilities is slightly larger, a factor of 2.5 (see Table 2), indicates that the change in permeability is not fully explained by free energy barriers.

To obtain a better estimate of the ammonia permeability, without the AWH bias affecting the dynamics, we ran additional shorter unbiased simulations for ammonia. For computational efficiency, the simulations were started from configurations sampled from the AWH-biased simulations where ammonia was inside of the pore; ammonia then typically leaves the pore within several nanoseconds. The permeabilities, computed using the calculated free energy together with pore exit times and probabilities (see sec:methods for details), are *p*_*d*_ = 0.7 ± 0.1 10^−14^ cm^3^ s^−1^ for TIP2;1 and 0.18 ± 0.04 10^−14^ cm^3^ s^−1^ for the double mutant. Thus, the *p*_*d*_ reduction due to the double mutation is a factor of 4 which, given the change in *p*_*d*_ values for water (see Table 2), corresponds to a increase in selectivity for water by a factor of 1.6. The calculated *p*_*d*_ value for TIP2;1 is lower than *p*_*d*_ for water, but higher than expected from the difference in maximum free energy barriers. It could be that the friction in the pore for ammonia is lower than for water. In addition, the absolute *p*_*d*_ values for ammonia could be affected by an approximation we make in calculating *p*_*d*_; that the attempt rate for ammonia to enter the pore is the same as for water. However, we expect this approximation to affect both TIP2;1 and the double mutant similarly and thus not affect the selectivity factor significantly.

Also in our previous analysis concerning the position of the H185 side chain and the amount of pore–water interactions, the CHARMM double mutant stood out. These factors thus seem important for observing a significant, albeit small, selective effect. A narrower pore in combination with an additional rigid interaction site decreases the number of ways for water to arrange favorably around a solute molecule. Therefore, the selectivity mechanism we propose in this case is that of decreased entropy of water in the pore compared to TIP2;1.

### Potential gating mechanism

**Figure 6:**
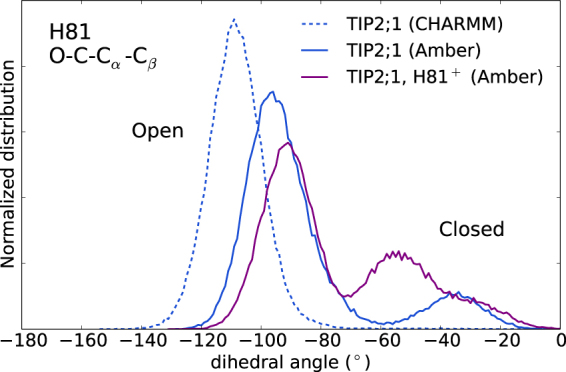
Distribution of H81 dihedral angle characterizing closing of the pore. The dihedral definition is indicated in the figure. Solid and dashed lines are for Amber and CHARMM, respectively. H81^+^ denotes that H81 is doubly protonated. Each histogram is based on one 1 *μ*s long trajectory. See Supplementary Fig. S2 for the corresponding trajectories.

In our simulations with Amber, we occasionally see the histidine H81 on the cytosolic end (*z* < 0) shift and “plug” the pore, which decreases the permeability by an order of magnitude and the pore radius down to less than 1 Å (see Fig. 3). In total, in all simulated systems, we observe four closing and three opening events. The closing times vary from 200 ns to more than 1 *μ*s (not observed). Thus, it is clear that these events are rare on the simulated timescale. The swinging motion of H81 is described well by the dihedral angle O-C-C_*α*_-C_*β*_ for which open corresponds to −94° and closed to −35° (±10°), see distributions in Fig. 6. For CHARMM the magnitude of this angle is on average larger than for Amber and closing is not observed.

This appears to be the same closing mechanism observed in simulations of human AQP4^18,19^ and AQP5^20^ where closing involved the conserved histidine corresponding to H81 here. And indeed, reports from both simulations and experiments show that aquaporins are gated channels^14,18–26^. For instance, grapevine TIP2;1 has been suggested to be gated by both pH^24^ and pressure^25^. The pH sensitivity was linked to a histidine located on loop D^24^, which is however not present in wheat TIP2;1 that we are studying here. The residues proposed to play a role in the gating motion are most commonly located at the cytosolic end of the pore. Interestingly however, different aquaporins display distinct closing mechanism, involving residues located in distant regions of the sequence: loop D in spinach aquaporin PIP2;1^21^, the N-terminus in yeast aquaporin Aqy1^22^ and here H81 located on loop B. Previously, it has been suggested that mammalian and plant aquaporin gating are distinct^18,27^. Our observations here rather demonstrate the presence of a gating mechanism common to humans and plants.

The disruption and reforming of a hydrogen bond between E24 with the adjacent serine S78 appears to be a telling indicator of the closing and opening events, respectively. For CHARMM, in contrast, this interaction is extremely stable; the minimum distance E24–S78 always staying below or close to 2.5 Å. A serine in AQP5 corresponding to S78 in TIP2;1 was previously simulated in a phosphorylated state in an attempt to reverse the closing event and open the pore^20^. In the present case, a consistent hypothesis is rather that phosporylation of S78 would promote the closed state since the added negative charge on S78 would be repelled by the negatively charged E24, increasing the distance between them.

The role of the cytosolic histidine in pH regulation of permeability was studied for AQP4 by protonating the histidine^18^. It was proposed that the conserved glutamic acid, here E24, could “trap” the histidine, preventing it from occluding the pore which would be consistent with the experimental finding for AQP4 that lower pH increases permeability. It was noted that experiments do not generally agree and that for plants permeability generally decrease with lowered pH. Here, we also protonated H81 of TIP2;1 and performed a 1 *μ*s long simulation. We do not observe this promoting an open, permeable state. For one monomer, H81 blocks the pore already during the first 200 ns of equilibration. Overall, the permeability is lowered. The dihedral angle distribution of H81 is shifted toward the closed state, see Fig. 6, and the free energy barrier between the open and closed state is lower. Thus, the protonation seems to make the closed state more accessible and transitions between opening and closing, faster. We would finally like to emphasize that longer simulations possibly in combination with enhanced sampling methods targeting the opening/closing events would be required to more accurately compare these different systems.

## Discussion and Conclusion

We have investigated the selectivity and permeability of the plant aquaporin TIP2;1 in a mutational study using MD simulations. To understand fine-tuned selectivity in aquaporins, we have focused on the challenging case of ammonia permeation. While TIP2;1 is known to be both ammonia and water permeable, according to experiments the double SF mutation I185H × G194C makes the channel select against ammonia, without sacrificing water permeability. Experiments further indicate that neither of the single mutations are sufficient to achieve this effect. Thus, simulating TIP2;1 and these mutants poses an interesting test case for which selectivity seems to arise from a cooperative effect between SF residues.

We have calculated water permeabilities for all the simulated systems, and the results show that all the mutations decrease the water permeability of the pore, to varying degrees. The greatest difference is seen for the CHARMM double mutant, where the permeability decreases by a factor of 2.5. We explain this effect by increased water–pore interactions and, when the I185H mutation is present, a narrower pore constriction. The G194C mutation does not add or change the interaction sites of the pore, but the induced small structural change influences also the orientation and dynamics neighboring SF residues, with measurable effects on free energies and permeabilities.

From simulations with ammonia we determined free energies and computed the effect of mutations on ammonia permeability. For the CHARMM double mutant, the permeability decreased by a factor of 1.6 more for ammonia than for water, consistent with increased selectivity for water. The accompanying change in water permeability demonstrates a trade-off between selectivity and permeability. The same system also showed a significant increase in water–pore hydrogen bonding in the SF region, which is likely the origin of increased selectivity in this case. This selectivity mechanism is consistent with previous results for AQP1, where a correlation between hydrophobicity and free energy barrier heights was demonstrated and explained by the disruption of water–pore interactions, during the passage of an apolar solute^6^. Conversely, these results for the double mutant show that ammonia permeation relies on the SF being relatively wide and hydrophobic.

This change in selectivity is however surprisingly small. Experiments measuring acidification rates showed that the double mutation decreased permeability by two orders of magnitude^10^. Modeling-wise, the protonation states of the histidines in the SF, which we have not experimented with in our simulations, could be an important factor. Alternatively, it could motivate running even longer simulation, allowing for some unknown, larger conformational change to take place, although we note that the resolution of the crystal structure is high, 1.18 Å.

The extensive amount of sampling in this work, up to 8 *μ*s of accumulated data per simulation setup, combined with the application of two force fields has enabled us to validate the simulated models on both short and longer timescales. For CHARMM, we see rare, structural changes involving the NPA region. For Amber, we observe gating-like motion of H81, closing the pore on the same timescale as the simulation. On shorter timescales, we have detected significant effects of mutations on both distributions and dynamics. In some cases, the difference between force fields were larger than differences between the mutants, showing that modeling is a main source of uncertainty. However, it is encouraging that simulation lengths and sampling methods have developed to the extent that we can map out subtle differences between systems with high accuracy, providing insight into biological mechanisms, as well as the means for validating and further improving modeling.

## Methods

### MD simulations

Simulations were performed using a development branch of GROMACS^28^, extended by an implementation of AWH^29^. We used the CHARMM36^30,31^ force field with CHARMM-modified TIP3P water^32^. Parameters for ammonia were generated using MATCH^33^. Protein and lipid virtual sites together with constraints on all bonds using LINCS^34^ and heavy hydrogens on ammonia when present, enabled us to use a 5 fs MD time step^35,36^. Redistributing the mass of the ammonia molecule, the three hydrogen masses were increased by 3 u and the nitrogen mass decreased by 9 u^37^, slows down internal angle vibrations. The temperature was kept at 310 K using the v-rescale thermostat^38^ and the pressure at 1 bar using *xy*-isotropic Berendsen pressure coupling^39^. Long-range electrostatics were calculated using particle mesh Ewald (PME)^40^ with a distance cutoff of 12 Å, Fourier space grid spacing 1.4 Å and interpolation order 4. Long-range Lennard-Jones interactions were calculated by switching the force to zero for atom distances 10–12 Å.

The Amber ff99SB-ILDN force field^41^ was used together with Berger united-atom lipids^42^, TIP3P water^43^ and Na^+^ and Cl^−^ ions. Ammonia was modeled with GAFF parameters^44^ generated using Antechamber^45^. We used GROMACS v.5.0.6 to generate the protein topology. Changes in MD parameters for Amber were as follows: for PME, we used a distance cutoff 10 Å, and a Fourier space grid spacing 1.25 Å; the Lennard-Jones cutoff 10 Å was handled by shifting the potential to zero; dispersion correction was applied for energy and pressure.

The crystal structure of TIP2;1 (PDB code 5I32) was embedded as a tetramer in a periodic POPC lipid bilayer which was generated using the CHARMM membrane builder^46^ and solvated in water with K^+^ and Cl^−^ ions added to obtain a concentration of 0.15 M. Mutations and tetrameric symmetry operations were applied using PyMOL^47^. Most histidines were protonated at sites predicted by the GROMACS tool pdb2gmx (using hydrogen network analysis): H63:N_*δ*_, H81:N_*δ*_, H214:${{\rm{N}}}_{\epsilon }$. H131 was protonated on ${{\rm{N}}}_{\epsilon }$ and the mutation H185 on N_*δ*_.

The system was energy minimized using the steepest descent algorithm until the maximum force was less than 500 kJ · mol^−1^ Å^−1^. Then it was equilibrated with MD by gradually releasing restraints as follows: NVT, heavy atoms restrained, 500 ps; NPT, heavy atoms restrained, 10 ns; NPT, backbone restrained, 20 ns; NPT, *C*_*α*_ restrained, 60 ns, NPT, no restraints 200 ns. Configurations from the equilibration were saved every 1 ns; for the following production runs, every 50 ps.

### Trajectory analysis

Generally in our analysis, the origin of the channel axis *z* is placed at the center of mass (COM) of the backbone of each monomer. We note that this happens to lie very close to (~0.3 Å along *z* from) the COM of the NPA residues. For the permeability calculation we for practical purposes used the center of geometry instead of the COM.

Pore radii were calculated using HOLE v.2.2.004^48^.

Hydrogen bonds were detected using the MDAnalysis^49^ hydrogen bond library, applying a donor-acceptor atom distance cutoff 3.5 Å and a donor-hydrogen-acceptor cutoff angle of 150° (where 180° is linear).

The osmotic permeability, *p*_*f*_ was calculated per pore using the collective diffusion method^13^. The net permeation *n*(*t*) was determined by time integration of ${\rm{d}}n={\sum }_{i}\,({z}_{i}(t)-{z}_{i}(t-{\rm{\Delta }}t))/L$, where *i* runs over the waters the pore during *t* − Δ*t* to *t* and the pore is defined by |*z*| < 9 Å = *L*/2 and *d*_*xy*_ ≤ 7 Å. Δ*t* = 50 ps, the time between trajectory frames. We get very similar results with Δ*t* = 5 ps. The full trajectory of 1 *μ*s was divided into ten, 100 ns, segments and analyzed separately. For each segment the mean square displacement 〈Δ*n*^2^(*τ*)〉 = 〈(*n*(*t* + *τ*) − *n*(*t*))^2^〉 was calculated for all pairs of times, for 0 < *τ* < 1000 ps. The diffusion coefficient, proportional to *p*_*f*_, was obtained as half the slope of 〈Δ*n*^2^(*τ*)〉, excluding *τ* ≤ 50 ps. The diffusion permeability was obtained simply by counting the number of permeation events per pore^50^.

VMD^51^ was used for analysis and trajectory visualization, including rendering of trajectory snapshots. The GROMACS tools and MDAnalysis^49^ libraries were used in the analysis scripts.

### Free energy calculations

The free energy along the pore axis for ammonia was calculated using AWH^52,53^. AWH is an enhanced sampling algorithm which, within a single simulation, adaptively estimates the free energy along a given reaction coordinate and applies a bias potential consistent with a chosen target distribution (often uniform). The free energy estimate and the bias are thus closely linked through the target distribution, such that initially when the uncertainty in the free energy is high and its estimate fluctuates a lot, the bias also changes rapidly. In this initial stage, the system will be pushed towards regions of phase space where the coordinate is under-sampled relative to the target distribution. The result is a back-and-forth motion of the coordinate which becomes increasingly diffusive as sampling proceeds and the free energy converges. Even when obtaining the free energy is difficult or infeasible to obtain because of reaction coordinate issues or because convergence requires several transitions across the predefined coordinate interval, AWH can be used as an explorational sampling tool^54,55^.

The input AWH needs is a sampling interval, the target distribution along the coordinate, the resolution of the added bias (expressed in terms of the force constant of a harmonic potential), the initial uncertainty of the free energy ${\hat{\epsilon }}_{0}$ (in *k*_*B*_*T*) and an estimate $\hat{D}$ of the diffusion constant along the coordinate The latter two sound like non-trivial parameters to set but as any adaptive biasing method, AWH needs some information related to the timescale of the system to optimally initialize the rate of change of the bias. Note that the values of $\hat{D}$ and ${\hat{\epsilon }}_{0}$ only affect the efficiency of the method and so does not have to in any way match their real values.

Here, for each equilibrated structure we applied four independent AWH bias potentials, one for each monomer, and simulate for 1 *μ*s. This typically yielded tens of transitions along each pore. Each bias acts on the center of mass *z* distance between an ammonia molecule and the backbone of one of the monomers. The sampling interval was *z* ∈ [−20, +20] Å, which was uniformly covered by *i* = 1, …, 237 harmonic potentials: ${V}_{i}(z)=\frac{1}{2}k{(z-{z}_{i})}^{2}$, where *k* = 10 kJ · mol^−1^ Å^−2^. To keep the solute closeby the pore entrance, the coordinate radial distance was restrained to stay below *d*_*xy*,max_ = 6 Å by adding a flat-bottom umbrella potential: $V({d}_{xy})=\frac{1}{2}k{({d}_{xy}-{d}_{xy,{\rm{\max }}})}^{2}$, for *d*_*xy*_ > *d*_*xy*,max_ and zero otherwise. The rate of change of each AWH bias was initialized by setting the initial average free energy error to ${\hat{\epsilon }}_{0}=\mathrm{3\ }{k}_{B}T$ and $\hat{D}=1\cdot {10}^{-2}$ Å^2^ ps^−1^. The order of magnitude value for $\hat{D}$ was found in^56^.

For water, we obtained free energies by simply counting waters in bins along *z* (in unbiased simulations without ammonia) and taking the logarithm of the counts.

Post-simulation, the average free energy of the free energy for each monomer was calculated as a self-consistent exponential average and error bars were obtained from jackknife errors as described in^53^.

### Ammonia permeability calculations

To compute the permeability of ammonia we ran free simulations, using as starting configurations frames sampled from AWH-biased trajectories where ammonia is in the pore. By combining results from these free simulations with the free energy profile, we can compute the crossing rate for ammonia and the permeability.

For the analysis we divided the pore into a lower section, *z*_*l*_ < *z* < *z*_0_, and an upper section *z*_0_ < *z* < *z*_*u*_ where *z*_0_ = 0 Å and the lower and upper boundaries, *z*_*l*_ and *z*_*u*_, were chosen such that the free energy *F*(*z*_*l*_) = *F*(*z*_*u*_) = 2.0 *k*_*B*_*T* relative to bulk water. We assume that the main contribution to the friction comes from *z*_*l*_ < *z* < *z*_*u*_. We then compute the one-way permeation rate (units s^−1^) from *z*_*l*_ to *z*_*u*_ as

1 $$r({z}_{l}\to {z}_{u})=k({z}_{l};{z}_{u})p({z}_{l}\to {z}_{u}),$$

where *k*(*z*_*l*_; *z*_*u*_) is the number of crossing events at *z*_*l*_ in the direction of *z*_*u*_ per unit time; and *p*(*z*_*l*_ → *z*_*u*_) is the probability of reaching *z*_*u*_ before returning to *z*_*l*_ when crossing at *z*_*l*_ in the direction of *z*_*u*_. We factorize the probability as *p*(*z*_*l*_ → *z*_*u*_) = *p*(*z*_*l*_ → *z*_0_)*f*(*z*_0_ → *z*_*u*_), where *f*(*z*_0_ → *z*_*u*_) is the fraction of molecules starting at *z*_0_ that exit the pore upward, at *z*_*u*_. With this decomposition we assume that velocities decorrelate quickly after crossing *z*_0_, which is reasonable since the crossing frequency at *z*_0_ is ~10^3^ higher than the exit frequency. Since our starting configurations are such that we mainly see ammonia exiting, and not entering, the pore, we apply the principle of detailed balance to reverse the crossing probability using the free energy, $p({z}_{l}\to {z}_{0})=p({z}_{0}\to {z}_{l}){e}^{F({z}_{l})-F({z}_{0})}$. We thus arrive at,

2 $$r({z}_{l}\to {z}_{u})=k({z}_{l};{z}_{u})p({z}_{0}\to {z}_{l}){e}^{F({z}_{l})-F({z}_{0})}f({z}_{0}\to {z}_{u}\mathrm{)}.$$

Directly measuring *k* for ammonia at a low concentration would require a lot of sampling, so we instead estimate *k* ≈ *ρk*_w_ exp(*F*_w_ − *F*), where *ρ* = *n*/*N* is the fraction of ammonia molecules, *k*_w_ is the crossing rate for water and *F*_w_ is the free energy for water. The mass difference between the two molecules is small, but this does assume that they interact similarly with pore-lining residues at the boundaries. In the limit of low ammonia concentrations, *ρ* can further be related to the number concentration *c* = *n*/*V* as *ρ* = *cv*_w_, where *v*_w_ is the volume of a water molecule. The permeability *p*_*d*_ is defined as the proportionality constant relating a concentration difference between two reservoirs to the resulting permeation rate, *r* = *p*_*d*_Δ*c*^57^. Assuming for convenience that the concentration is zero for *z* > *z*_*u*_, so that Δ*c* = *c*, and substituting our expression for *k* into Eq. (2), we obtain

3 $${p}_{d}\approx {v}_{{\rm{w}}}{k}_{{\rm{w}}}({z}_{l};{z}_{u}){e}^{{F}_{{\rm{w}}}({z}_{l})-F({z}_{l})}p({z}_{0}\to {z}_{l}){e}^{F({z}_{l})-F({z}_{0})}f({z}_{0}\to {z}_{u})$$

An expression using the downward rate *r*(*z*_*u*_ → *z*_*l*_) can be derived analogously. Note that for passive transport, as is the case here, the downward rate should match the upward rate.

We calculated *p*_*d*_ for both directions. Capturing all (re-)crossings required a time resolution of 0.01 ps. As a check we computed *p*_*d*_ for water through TIP2;1 and we recover the value of 1.0 10^−14^ cm^3^ s^−1^ obtained from counting permeation events (Table 2). For ammonia, the result is a *p*_*d*_ value of 0.7 ± 0.1 10^−14^ cm^3^ s^−1^ and 0.18 ± 0.04 10^−14^ cm^3^ s^−1^ for TIP2;1 and double mutant, respectively. The fairly large uncertainty arises from uncertainties in the relative free energies.

### Data availability

A representative subset of the datasets generated during and/or analysed during the current study are available at 10.5281/zenodo.1167656. The remaining datasets generated during and/or analysed during the current study are available from the corresponding author on reasonable request.

## Electronic supplementary material

Supplementary information
