# Supplementary material for: Permeability and ammonia selectivity in aquaporin TIP2;1: linking structure to function
Source: Sci Rep. 2018 Feb 14;8:2995. doi: 10.1038/s41598-018-21357-2 (PMC5813003; doi:10.1038/s41598-018-21357-2)
Supplement: Supplementary file 1 — Supplementary information [file 41598_2018_21357_MOESM1_ESM.pdf]

Supplementary information for "Permeability and ammonia selectivity in aquaporin TIP2;1: linking structure to function"

Viveca Lindahl[1*], Pontus Gourdon[2], Magnus Andersson[1], Berk Hess[1]

1 Department of Physics and Swedish e-Science Research Center, KTH Royal Institute of Technology, Science for Life Laboratory, Stockholm, Sweden
2 Department of Biomedical Sciences, University of Copenhagen, Denmark; Department of Experimental Medical Science, Lund University, Sweden

* vivecal@kth.se

Table S1. Summary of simulation data

Table 1. List of simulations

Forcefield	System	Type	Length	Comment	Excl.	n
CHARMM	TIP2;1	equilibration	200 ns			6
		unbiased	1 μs		1	
		unbiased	1 μs	$\Delta t = 2.5$ fs	1	
		biased, NH_3	1 μs			
	I185H×G194C	equilibration	200 ns			7
		unbiased	1 μs			
		unbiased	1 μs	$\Delta t = 2.5$ fs	1	
		biased, NH_3	1 μs		1	
	G194C	equilibration	200 ns			3
		unbiased	1 μs		1	
		biased, NH_3	1 μs			
	I185H	equilibration	200 ns			3
		unbiased	1 μs		1	
		biased, NH_3	1 μs			
Amber	TIP2;1	equilibration	200 ns			8
		unbiased	1 μs			
		unbiased	1 μs	cont. from 1 μs		
		biased, NH_3	1 μs			
		equilibration	200 ns	$H81^+$		4
		unbiased	1 μs	$H81^+$		
	I185H×G194C	equilibration	200 ns			8
		unbiased	1 μs			
		unbiased	1 μs	cont. from 1 μs		
		biased, NH_3	1 μs			
	G194C	equilibration	200 ns			4
		unbiased	1 μs			
	I185H	equilibration	200 ns			4
		unbiased	1 μs			

Summary of generated simulation data. The 200 ns equilibration run of each system precedes the other simulations of that system. Furthermore, although not listed here, each equilibration was preceded by a total of 90 ns of protein-constrained simulation time. The number of monomers, if any, that are excluded ("excl.") from averages based on the NPA stability criterion (defined in the main text) are also listed. The number of monomers, n, included in calculating averages μ for unbiased simulations (i.e. all but the excluded ones) are listed alongside the respective equilibration run.

Figure S1. Free energies including single mutants

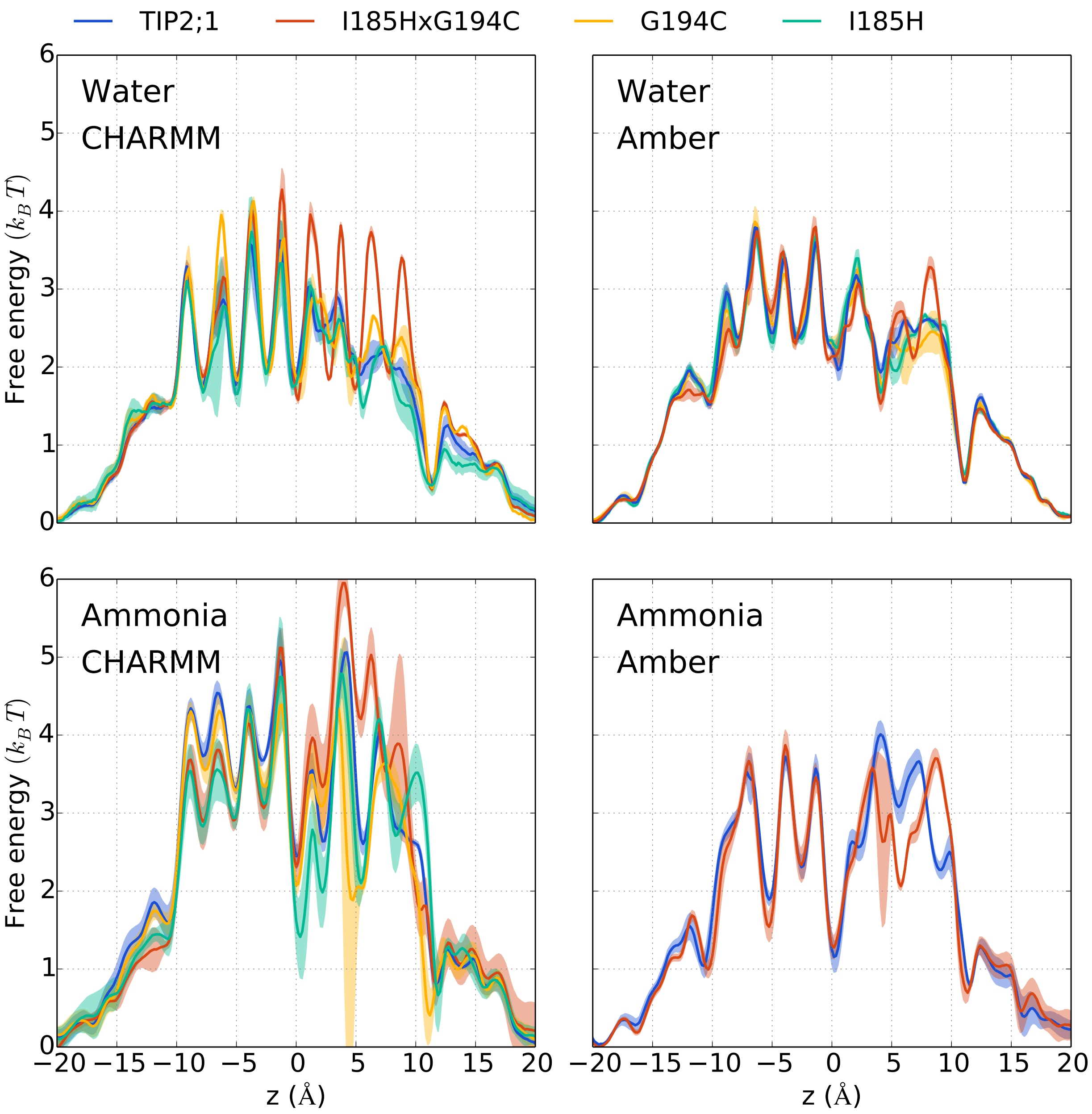

Figure 1: Free energies along z for ammonia and water. The error bars indicates estimated uncertainties of the average taken over the monomers. The larger error bars at at $z \approx 4$ Å in the ammonia profiles of CHARMM G194C and Amber I185H×G194C are due to rare sampling events of a side pocket in one of the monomers in each simulation. There is no ammonia data for the Amber single mutants.

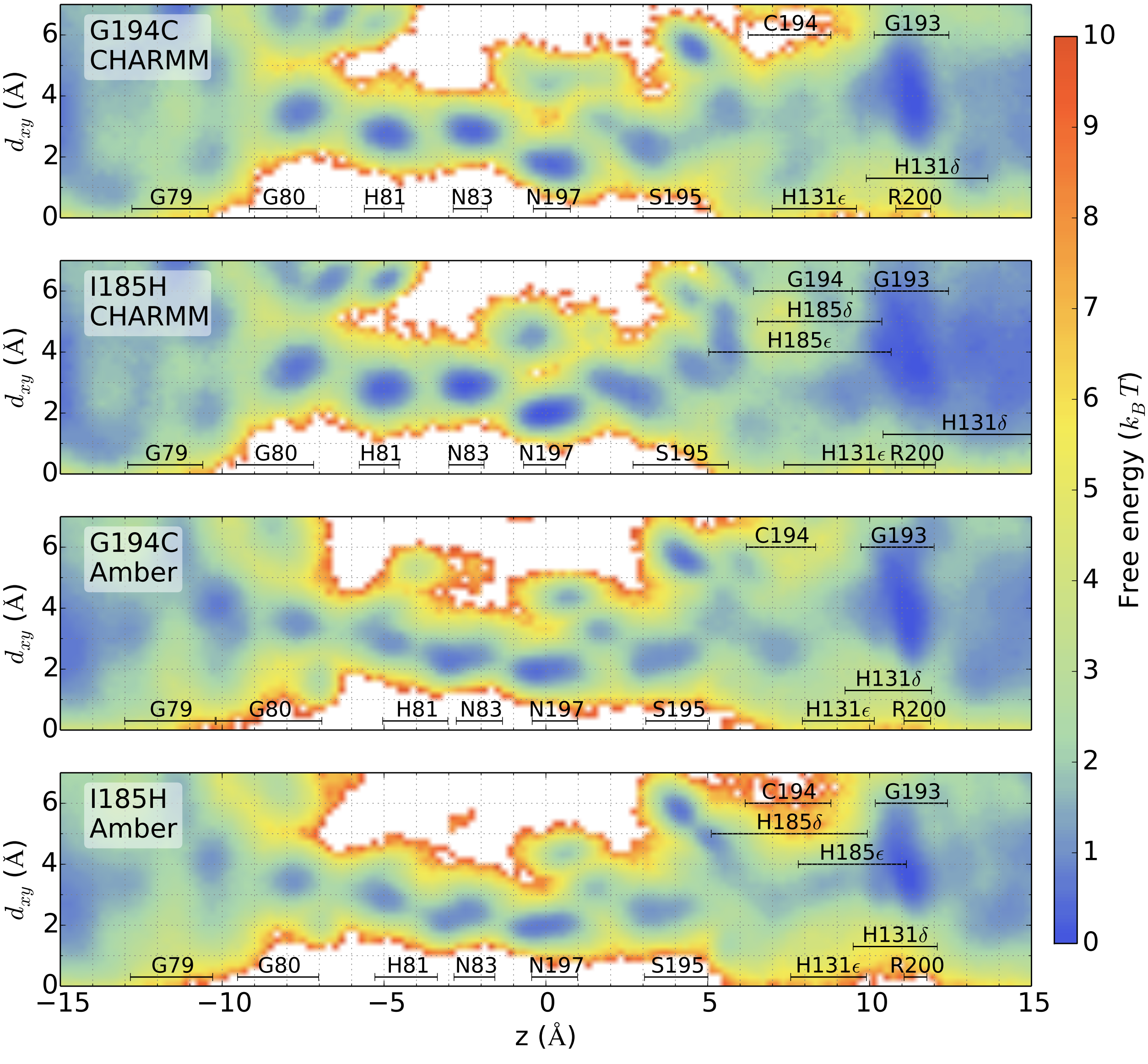

Figure 2: Free energy of water positions as a function of the distance along z and the radial distance from the channel COM, d_{xy} for the single mutants.

Figure S2. Histidine dihedral angle trajectories

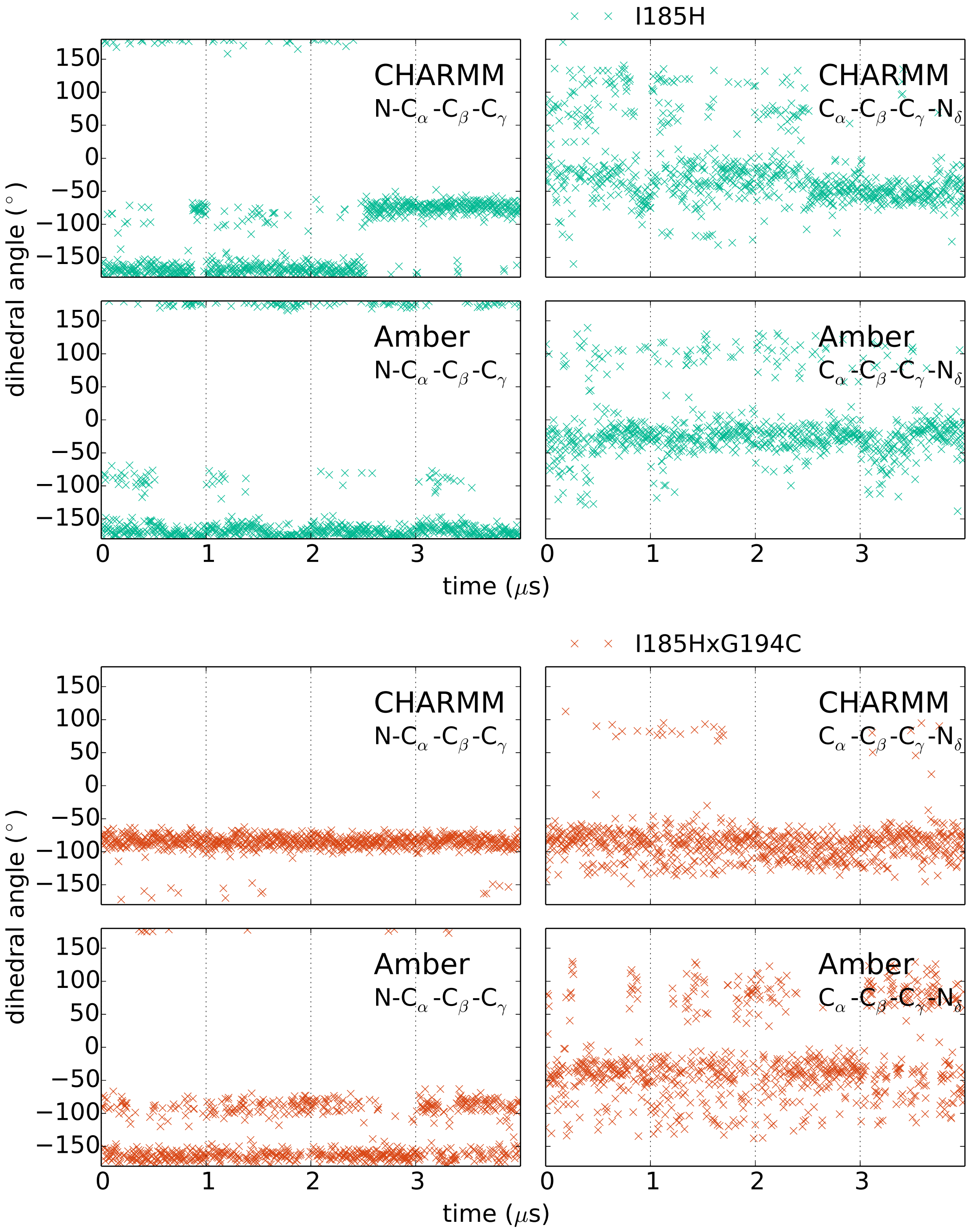

Figure 1: Trajectories of two H185 dihedral angles of mutants I185H and I185HxG194C. The dihedral definitions are indicated in the figure. The data is from one 1 μs long trajectory of each mutant. The trajectories of the four monomers have been concatenated so that times 0–1 μs corresponds to the first monomer, times 1–2 μs to the second monomer, etc.

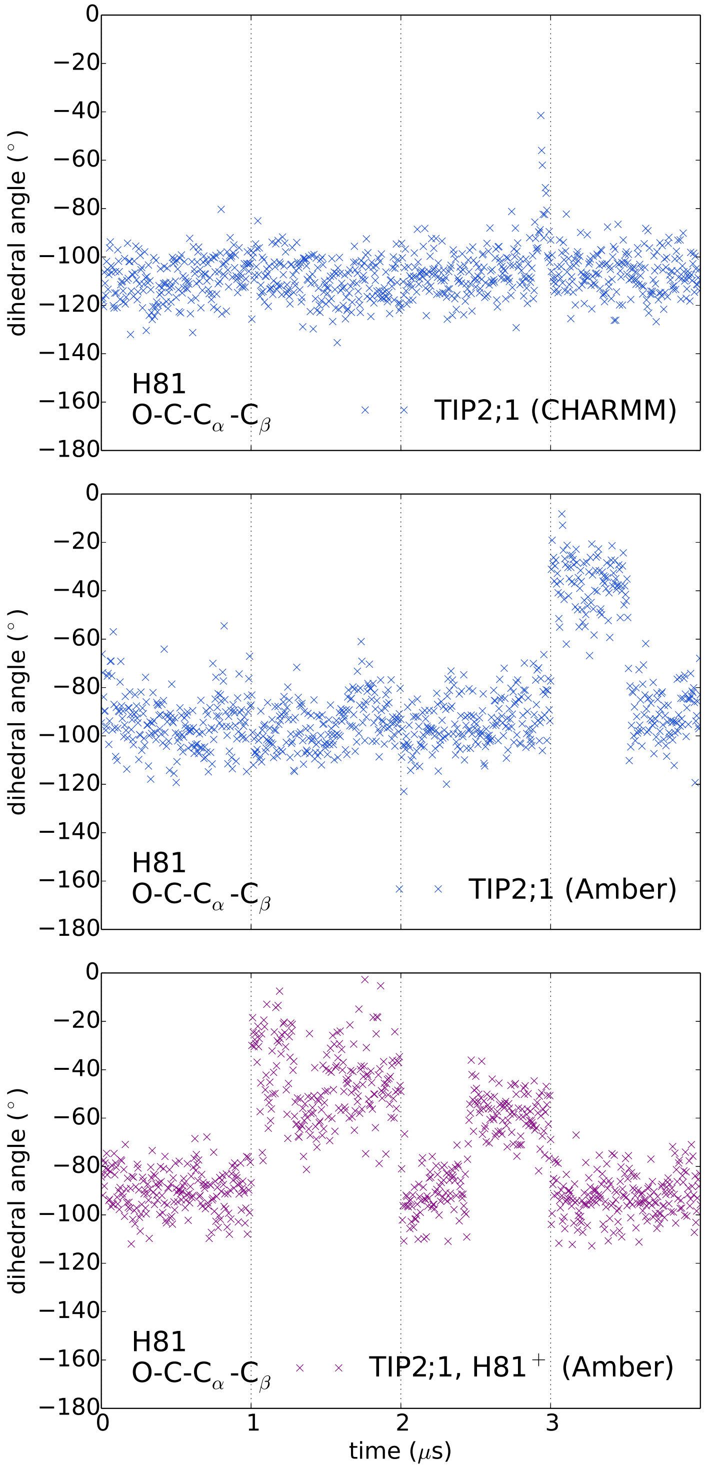

Figure 2: Trajectories of H81 dihedral angle characterizing closing of the pore. The dihedral definition is indicated in the figure. $H81^{+}$ denotes that H81 is doubly protonated. The data is from one 1 μs long trajectory of system. The trajectories of the four monomers have been concatenated so that times 0–1 μs corresponds to the first monomer, times 1–2 μs to the second monomer, etc.
